# Ten-year outcomes in first episode psychotic major depression patients compared with schizophrenia and bipolar patients

**DOI:** 10.1016/j.schres.2016.04.049

**Published:** 2016-10

**Authors:** M. Heslin, J.M. Lappin, K. Donoghue, B. Lomas, U. Reininghaus, A. Onyejiaka, T. Croudace, P.B. Jones, R.M. Murray, P. Fearon, G.A. Doody, P. Dazzan, T.J. Craig, C. Morgan

**Affiliations:** a King's College London, London, UK; b University of New South Wales, Sydney, Australia; c Nottinghamshire Healthcare NHS trust, UK; d Maastricht University, The Netherlands; e University of Dundee, Dundee, UK; f University of Cambridge, and Cambridgeshire and Peterborough NHS Foundation Trust, Cambridge, UK; g Trinity College, Dublin, Ireland; h University of Nottingham, Nottingham, UK; i National Institute for Health Research (NIHR) Mental Health Biomedical Research Centre at South London and Maudsley NHS Foundation Trust, UK

**Keywords:** Psychotic major depression, Depression, Psychosis, Outcomes, Prognosis

## Abstract

We aimed to investigate long-term outcomes in psychotic major depression patients compared to schizophrenia and bipolar/manic psychosis patients, in an incidence sample, while accounting for diagnostic change.

Based on Aetiology and Ethnicity in Schizophrenia and Other Psychoses (ÆSOP and ÆSOP-10), a first episode psychosis cohort was followed-up 10 years after first presentation. The Schedules for Clinical Assessment in Neuropsychiatry, WHO Life Chart and Global Assessment of Functioning were used to assess clinical, social and service use outcomes.

Seventy-two PMD patients, 218 schizophrenia patients and 70 psychotic bipolar disorder/mania patients were identified at baseline. Differences in outcome between PMD and bipolar patients based on baseline and lifetime diagnosis were minimal. Differences in clinical, social and service use outcomes between PMD and schizophrenia were more substantial with PMD patients showing better outcomes on most variables. However, there was some weak evidence (albeit not quite statistically significant at p < 0.05) based on lifetime diagnoses that PMD patients were more likely to attempt suicide (OR 2.31, CI 0.98–5.42, p0.055) and self-harm (OR 2.34, CI 0.97–5.68, p0.060).

PMD patients have better social and service use outcomes compared to people with schizophrenia, but may be more likely to attempt suicide or self-harm. This unique profile is important for clinicians to consider in any risk assessment.

## 1 Introduction

Major depression with psychotic features, also known as Psychotic Major Depression (PMD), is defined by ICD–10 (WHO, 1993) as a depressive disorder with the addition of delusions, hallucinations or depressive stupor. A systematic review and meta-analysis by Kirkbride et al. (2012) reported a pooled incidence for PMD in England of 5.3 (95% CI 3.7–7.6) per 100,000 person years. This was compared with 3.7 per 100,000 person years (95% CI 3–4.5) for bipolar with psychotic symptoms and 15.2 per 100,000 person years (95% CI 11.9–19.5) for schizophrenia. These results suggest that PMD is less common than schizophrenia, but more common than bipolar disorder. Despite these incidence rates, PMD is a largely under-researched disorder (Crebbin et al., 2008).

Many studies have investigated the long-term course of illness and outcomes in psychosis (Ciompi, 1980, Harrison et al., 2001, Hill et al., 2012, Takei et al., 1998). However, studies have less often included outcomes on PMD patients (Ciccone and Racy, 1975, Schimmelmann et al., 2005, DelBello et al., 2003, Bromet et al., 1996) and importantly, few have compared outcomes in people with PMD to outcomes in other major psychotic diagnostic groups such as schizophrenia and bipolar patients. Further, many studies which include outcomes for PMD are based on prevalence samples or samples of only inpatients, both of which are biased towards those with longer duration and more severe illness (Cohen and Cohen, 1984) and consequently may give a distorted picture of long-term prognosis.

The four studies which have to date examined outcomes in PMD patients in incidence samples (Crebbin et al., 2008, Amin et al., 1999, Baldwin et al., 2005, Whitty et al., 2005) were conducted over a relatively short period of time (6 months–4 years); therefore, knowledge of longer-term outcomes is limited. While, three of these studies examined diagnostic stability only (Amin et al., 1999, Baldwin et al., 2005, Whitty et al., 2005). Crebbin et al. (2008) also reported some clinical and service use outcomes. They found that there was a similar percentage of deaths in the year after first presentation in the people with PMD (9.5%, n10/105) and schizophrenia (9.6%; n7/73). They also reported no difference in number of admissions or admission days between those with PMD and those with schizophrenia, but more use of compulsory admissions in schizophrenia patients. Although the authors state that diagnosis was stable in PMD at 87%, this is contrary to findings in other studies (65% (Amin et al., 1999), 73% (Whitty et al., 2005), < 50% (Heslin et al., 2015)). Based on these studies, accounting for diagnostic stability is important for outcome research in PMD patients who change diagnosis may have different outcomes compared to those who start with that diagnosis and retain it over time.

### 1.1 Aims of the study

Given the paucity of information on long-term outcomes for PMD patients in less biased samples, we aimed to examine long term (10 year) outcomes in people with PMD, while improving on the methodological limitations of previous research by studying an incidence sample (the ÆSOP study), and accounting for diagnostic change. Outcomes in people with PMD were compared to outcomes for schizophrenia and bipolar/manic psychosis patients. Specifically, we chose to investigate the following aspects of outcome in PMD patients: clinical outcomes (symptoms, course of illness, suicide attempts and self-harm); social outcomes (disability, employment, relationship status, close confidants and time in prison); and service use outcomes (days hospitalised and compulsory admissions).

## 2 Methods

This paper is based on the ÆSOP-10 study which is fully described in Morgan et al. (2014) In brief, ÆSOP-10 is a 10 year follow-up of a cohort of people with a first episode of psychosis. The original cohort was identified from all inpatient and outpatient mental health services in two well defined catchment areas in the UK (Kirkbride et al., 2006). At baseline, detailed information was collected to enable re-contact for all patients. We aimed to trace, re-contact and re-interview all patients at approximately 10 years. Patients were contacted via current mental health services, if in contact with services, by inviting them to participate through their clinical teams. For those not in contact with services, letters were sent to their last known address inviting them to participate. Non-responders were sent a further letter two weeks later with a maximum of three visits to the address if needed to make initial contact. For those believed to have moved address, we sought to make contact and invite them to participate through their GP if known.

### 2.1 Measures

At baseline, data on demographics (age, gender, ethnicity, centre, place of birth) were collected using the Medical Research Council Socio-demographic Schedule (Mallett, 1997). The Schedules for Clinical Assessment in Neuropsychiatry (SCAN version 2 (WHO, 1994)) was used to elicit symptom-related data at the time of presentation. Symptom data plus all available clinical information (excluding diagnosis) was used to assign ICD-10 (WHO, 1993) psychotic diagnoses within consensus meetings involving the research team. These meetings involved at least one senior psychiatrist. Diagnosis was made as soon as possible after first contact (generally within a few weeks). Diagnoses were made blind to ethnicity and diagnosis from the clinical notes.

A range of measures were used to collect data at follow-up. Relevant to this paper are the SCAN, the WHO Life Chart and the Global Assessment of Functioning (GAF). The SCAN was repeated where interview with patients were possible, and completed in relation to the preceding month. An extended version (detailed in Morgan et al. (2014)) of the WHO Life Chart (Harrison et al., 2001, Sartorius et al., 1996, Burns et al., 1999) was completed for each patient using where possible, clinical interviews with patients and information from treating clinicians plus clinical notes, to map course of illness and symptom history. The Life Chart collates information on course of illness and three key areas of outcome: clinical; social; and service use. Items from the Life Chart relevant to this paper were: course of illness (episodic, continuous or neither); occurrence of suicide attempts and self-harm; relationship status, employment status, presence of a close confidant and whether the person spent any follow-up time in prison; and number of days as an inpatient and ever compulsorily admitted. Suicide attempts were defined as a deliberate act of self-harm with the intention of ending one's life. If there was any doubt about the intention, then it was rated as self-harm. Self-harm was defined as intentional injury to one's body. If there was any doubt about whether something was deliberate, it was not counted. The split GAF was used to characterise overall symptomatology and function in the month prior to follow-up (Harrison et al., 2001, adapted from Endicott et al., 1976) based on presentation at follow-up: the GAF symptom scale; and the GAF disability scale. Higher GAF scores indicate fewer symptoms or a better level of functioning. Information from the SCAN at follow-up and Life Chart were used to determine lifetime diagnosis using the consensus approach as at baseline, and blind to ethnicity and baseline diagnosis.

### 2.2 Ethics

Full ethical approval for all aspects of the follow-up was provided by the local research ethics committees in South East London and Nottingham. All researchers had substantive or honorary contracts with either the South London and Maudsley National Health Service (NHS) Foundation Trust or the Nottingham Healthcare NHS Trust, the primary participating service providers.

### 2.3 Analyses

All data were analysed using STATA (version 11; StataCorp, 2009). Data were described using means and standard deviations, medians and interquartile ranges or frequencies and percentages as appropriate. Outcomes for PMD patients were compared with outcomes for bipolar disorder and schizophrenia patients. Categorical outcomes were analysed using logistic regression. Continuous outcomes were analysed using bootstrap regression (1000 replications) to account for the skewed nature of the data (Kielhorn and Graf vonSchulenberg, 2000). Bootstrap regression analyses produce the same coefficients are interpreted in the same way as linear regressions but produce more robust confidence intervals.

## 3 Results

A total of 557 first episode patients were identified at baseline. Data presented here are based on the incidence sample (n, 505) collected over the first 2 years (excluding: non-incidence patients collected for the brain imaging component of the study; patients oversampled in the 3rd year in order to increase the numbers for the ethnicity component of the study; and patients excluded post baseline). Data presented here are for the PMD, schizophrenia and bipolar disorder/mania patients only (n360) (i.e., excluding delusional disorder, schizoaffective disorder, acute & transient psychoses, drug induced psychoses and psychoses NOS).

### 3.1 Sample characteristics

Of the 360 patients included here, 72 had a baseline diagnosis of PMD, 218 had a diagnosis of schizophrenia, and 70 had a diagnosis of bipolar disorder or mania with psychotic symptoms at baseline. Table 1 describes the demographics of these three groups and the sample overall. Of the 360 patients of interest, 24 patients had died and 23 patients had moved abroad. Two hundred and sixty eight had some follow-up data at 8–12 years (54 PMD patients, 161 schizophrenia patients and 53 bipolar/mania patients). Therefore only 45 patients were lost to follow-up (12.5%). Differences in the proportion followed up by diagnostic group were compared, and found not to differ (Chi2 (2df) 0.11, p 0.946).

### 3.2 Outcomes by baseline diagnosis

Outcomes are described only for the core analytic sample (excluding those who died, moved abroad or were lost to follow-up as described by Morgan et al. (2014). Table 2 describes the outcomes by baseline diagnosis for PMD, schizophrenia and bipolar/mania patients. The table shows that the only differences between PMD patients and bipolar patients were as follows: episodic course of illness which was less likely in PMD patients (OR 0.16, CI 0.06 to 0.40, p < 0.01); GAF disability score at follow-up which was lower in PMD patients (indicating worse functioning, coefficient − 10.85, CI − 18.66 to − 3.04, p < 0.01); and having been compulsorily admitted, which was far less likely to happen to PMD patients (OR 0.22, CI 0.08 to 0.59, p < 0.01).

There were more differences between PMD and schizophrenia patients. Compared with those with schizophrenia, PMD patients had a higher GAF symptom score at follow-up (indicating better functioning, coefficient 14.25, CI 8.13 to 20.37, p < 0.01); were more likely to have an episodic course of illness (OR 3.02, CI 1.46 to 6.25, p < 0.01); had a higher GAF disability score at follow-up (coefficient 11.22, CI 4.78 to 17.65, p < 0.01); were less likely to be employed for less than 25% of the follow-up (OR 0.34, CI 0.16 to 0.70, p < 0.01); were more likely to be in a relationship over the follow-up (OR 4.14, CI 1.95 to 8.78, p < 0.01), were less likely to spend time in prison (OR 0.22, CI 0.05 to 0.97, p < 0.05); were less likely to be admitted compulsorily (OR 0.37, CI 0.18 to 0.76, p < 0.01); and spent fewer inpatient days in hospital (coefficient − 161.98, CI − 272.25 to − 51.70, p < 0.01).

### 3.3 Outcomes by lifetime diagnosis

Table 3 describes the outcomes by lifetime diagnosis for PMD, schizophrenia and bipolar/mania patients as well as comparisons between the PMD group and schizophrenia group, and the PMD and bipolar group. Data on diagnostic change are presented elsewhere (Heslin et al., 2015). In brief, of the 403 with baseline and lifetime diagnostic data, 15 changed from a baseline diagnosis of PMD to a follow-up diagnosis of schizophrenia, and seven changed from a baseline diagnosis of PMD to a lifetime diagnosis of bipolar disorder. Eight changed from schizophrenia to PMD, and two changed from bipolar to PMD. In terms of comparisons between PMD and BP patients, similarly to the baseline analyses, PMD patients had a lower GAF disability score at follow-up (indicating worse functioning, coefficient − 10.16, CI − 18.99 to − 1.33, p < 0.05); and were less likely to have been compulsorily admitted (OR 0.32, CI 0.12 to 0.84, p < 0.05). GAF symptom score became significantly different between the groups with PMD patients having a lower score (and therefore worse symptoms, coefficient − 8.77, CI − 16.86 to − 0.69, p < 0.05). PMD patients were still less likely to have an episodic course of illness but this was not statistically significant (OR 0.44, CI 0.18 to 1.07, p 0.071).

Comparisons between PMD and schizophrenia patients again revealed a large number of differences. Compared with those with schizophrenia, PMD patients had a higher GAF symptom score at follow-up (coefficient 11.40, CI 4.17 to 18.62, p < 0.01); were more likely to have an episodic course of illness (OR 7.71, CI 3.51 to 16.92, p < 0.01); had a higher GAF disability score at follow-up (coefficient 12.78, CI 4.82 to 20.73, p < 0.01); were less likely to be employed for less than 25% of the follow-up (OR 0.28, CI 0.12 to 0.65, p < 0.01); were more likely to be in a relationship over the follow-up (OR 4.79, CI 2.12 to 10.85, p < 0.01); were less likely to be admitted compulsorily (OR 0.34, CI 0.15 to 0.75, p < 0.05); and spent fewer inpatient days in hospital (coefficient − 243.05, CI − 337.01 to − 149.09, p < 0.01). The finding that PMD patients were much less likely to go to prison over the follow-up was no longer evident (OR 0.33, CI 0.07–1.45, p > 0.05); and there was new evidence that PMD patients were more likely to have close confidants (OR 9.21, CI 2.01 to 42.19, p < 0.01). Additionally, there was some weak evidence (albeit not quite statistically significant at p < 0.05) that PMD patients were more likely to attempt suicide compared with schizophrenia patients (OR 2.31, CI 0.98 to 5.42, p 0.055); and that PMD patients were more likely to self-harm (OR 2.34, CI 0.97 to 5.68, p 0.060).

### 3.4 Post hoc analyses

Following the finding that PMD patients had better social outcomes compared with schizophrenia but were more likely to self-harm or attempt suicide, post-hoc exploratory analyses were conducted to test whether those who self-harmed or attempted suicide had worse social outcomes than those who did not. There was some weak evidence (albeit not quite statistically significant at p < 0.05) based on the baseline diagnosis that those who attempted suicide were less likely to have a close confidant (OR 0.19, CI 0.03–1.20, p 0.077). Based on the lifetime diagnoses, there was some weak evidence (not quite statistically significant at *p* < 0.05) that PMD patients who self-harmed were more likely to work for more than 25% of the follow-up (OR 8.57, CI 0.83–89.04, p 0.072).

## 4 Discussion

Findings in this paper highlight that people with PMD have better social and service use outcomes compared with people with schizophrenia, but appear more likely to attempt suicide or self-harm. Outcomes for people with PMD are similar to those for people with bipolar/mania. Further, important differences between diagnostic groups were detected when accounting for diagnostic change.

Despite addressing methodological limitations of previous work (using incidence sample while accounting for diagnostic change), these results are mostly consistent with previous research on outcomes in PMD patients compared to schizophrenia patients even though it is mostly based on non-incidence samples. This previous research suggests that compared to schizophrenia patients: PMD patients have a more episodic course of illness (92% versus 16%; Opjordsmoen, 1989), attempt suicide more (42% versus 27%; Radomsky et al., 1999) self-harm more (33% versus 18%, p < 0.01; Crebbin et al., 2008), have better employment outcomes (Bromet et al., 1996, Opjordsmoen, 1989, Jager et al., 2005, Tsuang and Coryell, 1993, Sands and Harrow, 1999) have more stable relationships (Jager et al., 2005), and have better social contacts (Opjordsmoen, 1989, Tsuang and Coryell, 1993). Results from this study are consistent with these findings. In terms of service use, the majority of studies show that PMD patients have better inpatient outcomes compared with schizophrenia patients (less hospitalisations, lower scores on hospital outcomes, less continuous hospitalisations (Jager et al., 2005, Bromet et al., 1996, Tsuang and Coryell, 1993, Sands and Harrow, 1999, Craig et al., 1997, Craig et al., 2000). However, some studies report worse inpatient outcomes in PMD patients (more patients admitted, more hospitalisations (Crebbin et al., 2008, Opjordsmoen, 1989, Stephens, 1982). This study is consistent with the former as there were less inpatient days for the PMD patients and less compulsory admissions.

Previous research on outcomes in PMD patients compared with patients with psychotic bipolar disorder have reported a range of non-significant differences or conflicting findings in a range of domains (Bromet et al., 1996, Welner et al., 1977, Aronson et al., 1987, Aronson et al., 1988, Winokur et al., 1992). This study is consistent with these findings as there were minimum differences between the groups.

### 4.1 Limitations

As with all research, a number of limitations should be borne in mind when considering the findings. Firstly, although loss to follow-up was small considering the length of the study, this could have led to biased results, perhaps by either more severe or less severe patients being lost to follow-up. However, there were no differences in follow-up rates between the diagnostic groups. A further limitation is the simplification of outcomes. Relationship status was categorised into ‘in a relationship’ versus ‘single/divorced/separated’. This is an over simplification of a very complex phenomenon. It assumes that the quality of all relationships is equivalent and that the experience of being divorced is the same as being single or separated. Nonetheless, it provides useful information about whether a person has a partner as a potential form of social support. The recording and definitions of suicide attempts and self-harm are also relatively crude; further investigation is warranted of the nature and circumstances surrounding such events. Further, the cross-sectional nature of some of the data limits any causal links, e.g. with the finding that PMD patients had better social outcomes compared with schizophrenia but were more likely to self-harm or attempt suicide, both variables were obtained using the WHO Life Chart assessed at 10 years without accounting for or noting of temporality. Therefore, we cannot tell which preceded which.

Finally, we cannot be sure whether lifetime diagnosis was influenced by factors beyond strict operational criteria (i.e. did outcomes influence what diagnosis was given). Lifetime diagnosis is often assumed to be more reliable and accurate than initial diagnosis as when patients present to services with a first episode of psychosis, the clinical picture can be confusing: patients may be experiencing psychotic symptoms, mood symptoms, confusion, distress, and it takes some time for the clinical diagnosis to become clearer. However, information on outcomes over time may well become (consciously or not) the basis for follow-up diagnosis. For example, people who self-harm and/or attempt suicide may be more likely to get a follow-up diagnosis of PMD, and less likely to get a follow-up diagnosis of schizophrenia. Further, as not all cases agreed to be interviewed at follow-up, some diagnoses had to be made on the basis of clinical notes. This may have introduced some bias around what clinicians choose to record and omit. This information bias also applies to assessment of some of the outcomes (e.g. self-harm) which would only be known about had clinicians found out, and recorded these events in the notes. Although these are clearly problems, it is less clear how these limitations might be overcome in future research.

### 4.2 Clinical implications

Based on lifetime diagnoses, PMD patients are around twice as likely as schizophrenia patients to attempt suicide, with around a third (31%) of patients attempting suicide at some point within the first ten years following first episode of psychosis (although this needs to be viewed with caution given the small sample size and consequently imprecise estimates of effect size). This is compared to 14.5% in a sample of depressed patients over 5 years (Holma et al., 2010). This highlights the need for clinicians to be extra vigilant of potential suicidal behaviour in PMD patients compared to schizophrenia patients, but also highlights the need to explore for the presence of psychotic symptoms in all depressive illness. However, PMD patients were hospitalised less and had comparatively good social outcomes. This contradiction is important for clinicians to bear in mind in any risk assessment. Further, we have identified an important subgroup of patients with a different emphasis of need. People with PMD are less likely to need help with improving social outcomes, but may need additional support to disclose and manage self-harm behaviour.

### 4.3 Future research

Although we have covered some key areas of outcome in this study (employment, social isolation (as indicated by relationship status and presence of a close confidant) and prison time), there are other important domains of outcome – such as poverty and housing (Warner, 2008) – that are beyond the scope of the data presented here. These key areas also need to be investigated in people with PMD. Further, a better understanding of the incongruous findings of better social and service outcomes but worse self-harm and suicide is needed.

## Conflict of interests

RM has received speaker honoraria from Janssen, Astra-Zeneca, Lilly, BMS and Roche. PJB is a member of scientific advisory boards for Roche and Otsuke. Other authors have nothing to disclose.

## Contributors

MH was responsible for the analysis and interpretation of the study and drafted the initial manuscript. TC, PBJ, RMM, PF, GAD, PD and CM were all principle investigators and were responsible for creation and design of the larger ÆSOP study. JML, KD, BL, UR, AO and TJC were all involved in study design and interpretation of results. All authors contributed to and have approved the final manuscript.

## Funding body agreements and policies

This work was supported by the UK Medical Research Council (ref. G0500817) and the Department of Health through the National Institute for Health Research (NIHR) Specialist Biomedical Research Centre for Mental Health award to the South London and Maudsley NHS Foundation Trust (SLaM) and the Institute of Psychiatry at King's College London. This paper represents independent research part funded by the National Institute for Health Research (NIHR) Biomedical Research Centre at South London and Maudsley NHS Foundation Trust and King’s College London. The views expressed are those of the author(s) and not necessarily those of the NHS, the NIHR or the Department of Health.

## Figures and Tables

**Table 1:** Baseline demographics of full sample and by diagnostic groups.

Age	PMD (n72)	Schizophrenia (n218)	Bipolar (n70)	Overall sample (n360)
	Median (IQR)	Median (IQR)	Median (IQR)	Median (IQR)
	32.50 (25–41)	29.00 (22–35)	27.00 (23 − 33)	29.00 (23–36)
	N (%)	N (%)	N (%)	N (%)
Study centre:				
London	35 (48.6)	151 (69.3)	44 (62.9)	230 (63.9)
Nottingham	37 (51.4)	67 (30.7)	26 (37.1)	130 (36.1)
Gender:				
Male	36 (50.0)	140 (64.2)	33 (47.1)	209 (58.1)
Female	36 (50.0)	78 (35.8)	37 (52.9)	151 (41.9)
Ethnicity:				
White British	37 (51.4)	81 (37.2)	27 (38.6)	145 (40.3)
African-Caribbean	8 (11.1)	61 (28.0)	14 (20.0)	30 (8.3)
Black African	7 (9.7)	33 (15.1)	11 (15.7)	83 (23.1)
White Other	4 (5.6)	22 (10.9)	4 (5.7)	51 (14.2)
Asian	7 (9.7)	10 (4.6)	6 (8.6)	23 (6.4)
Other (all)	9 (12.5)	11 (5.1)	8 (11.4)	28 (7.8)
Place of birth:				
UK	50 (69.4)	148 (69.8)	53 (76.8)	251 (71.1)
Non-UK	22 (30.6)	64 (30.2)	16 (23.2)	102 (28.9)

IQR = interquartile range.

**Table 2:** Comparison of outcomes by baseline diagnosis.

	Outcomes			Comparisons	
	PMD	SZ	BP	PMD vs. SZ	PMD vs. BP
Clinical outcomes					
	Median (IQR)	Median (IQR)	Median (IQR)	Beta coefficient (CI)	Beta coefficient (CI)

Global assessment of functioning — symptoms	71.5 (58.5–80.5)	55 (42–65)	79 (65–87)	14.25 (8.13 to 20.37)⁎⁎	− 4.80 (− 12.27 to 2.68)
	n (%)	n (%)	n (%)	OR (CI)	OR (CI)
Course of illness					
Not episodic (continuous/neither)	31 (63.3)	125 (83.9)	10 (21.7)	–	–
Episodic	18 (36.7)	24 (16.1)	36 (78.3)	3.02 (1.46 to 6.25)⁎⁎	0.16 (0.06 to 0.40)⁎⁎
Attempted suicide					
No	35 (79.6)	119 (83.8)	36 (83.7)	–	–
Yes	9 (20.5)	23 (16.2)	7 (16.3)	1.33 (0.56 to 3.14)	1.32 (0.44 to 3.94)
Self-harmed					
No	37 (80.4)	121 (86.4)	38 (88.4)	–	–
Yes	9 (19.6)	19 (13.6)	5 (11.6)	1.55 (0.65 to 3.71)	1.85 (0.57 to 6.04)

Social outcomes					
	Median (IQR)	Median (IQR)	Median (IQR)	Beta coefficient (CI)	Beta coefficient (CI)

Global Assessment of functioning — disability	62 (50–75)	47 (40–60)	80 (64–86)	11.22 (4.78 to 17.65)⁎⁎	− 10.85 (− 18.66 to − 3.04)⁎⁎
	n (%)	n (%)	n (%)	OR (CI)	OR (CI)
Employment status:					
Employed 25–100%	20 (50.0)	34 (25.2)	17 (47.2)	–	–
Employed 0–25%	20 (50.0)	101 (74.8)	19 (52.8)	0.34 (0.16 to 0.70)⁎⁎	0.89 (0.36 to 2.20)
Main relationship status:					
Single/divorced/separated	19 (48.7)	110 (79.7)	23 (56.1)	–	–
In a relationship	20 (51.3)	28 (20.3)	18 (43.9)	4.14 (1.95 to 8.78)⁎⁎	1.35 (0.56 to 3.24)
Close confidant:					
No	8 (28.6)	37 (46.3)	6 (25.0)	–	–
Yes	20 (71.4)	43 (53.8)	18 (75.0)	2.15 (0.85 to 5.45)	0.83 (0.24 to 2.87)
Went to prison:					
No	43 (95.6)	113 (82.5)	40 (93.0)	–	–
Yes	2 (4.4)	24 (17.5)	3 (7.0)	0.22 (0.05 to 0.97)⁎	0.62 (0.10 to 3.91)

Service use outcomes					
	Median (IQR)	Median (IQR)	Median (IQR)	Beta coefficient (CI)	Beta coefficient (CI)

Total number of days hospitalised	63.5 (19.5–181.6)	107 (25–275)	79 (24–182)	− 161.98 (− 272.25 to − 51.70)⁎⁎	− 74.44 (− 235.21 to 86.34)
	n (%)	n (%)	n (%)	OR (CI)	OR (CI)
Ever admitted compulsorily:					
No	23 (54.8)	37 (30.8)	8 (21.1)	–	–
Yes	19 (45.2)	83 (69.2)	30 (79.0)	0.37 (0.18 to 0.76)⁎⁎	0.22 (0.08 to 0.59)⁎⁎

OR = odds ratio; CI = 95% confidence interval; IQR = interquartile range; PMD = psychotic major depression; SZ = schizophrenia; BP = bipolar disorder.

⁎ p < 0.05.

⁎⁎ p < 0.01.

**Table 3:** Comparison of outcomes by lifetime diagnosis.

	Outcomes			Comparisons	
	PMD	SZ	BP	PMD vs. SZ	PMD vs. BP
Clinical outcomes					

	Median (IQR)	Median (IQR)	Median (IQR)	Beta coefficient (CI)	Beta coefficient (CI)

Global assessment of functioning — symptoms	65 (57–79)	55 (41–65)	78 (65–87)	11.40 (4.17 to 18.62)⁎⁎	− 8.77 (− 16.86 to − 0.69)⁎
	n (%)	n (%)	n (%)	OR (CI)	OR (CI)
Course of illness					
Not episodic (continuous/neither)	16 (44.4)	148 (86.1)	14 (25.9)	–	–
Episodic	20 (55.6)	24 (14.0)	40 (74.1)	7.71 (3.51 to 16.92)⁎⁎	0.44 (0.18 to 1.07)
Attempted suicide					
No	22 (68.8)	137 (83.5)	42 (82.4)	–	–
Yes	10 (31.3)	27 (16.5)	9 (17.7)	2.31 (0.98 to 5.42)	2.12 (0.75 to 5.99)
Self-harmed					
No	26 (74.3)	142 (87.1)	42 (85.7)	–	–
Yes	9 (25.7)	21 (12.9)	7 (14.3))	2.34 (0.97 to 5.68)	2.08 (0.69 to 6.25)

Social outcomes					
	Median (IQR)	Median (IQR)	Median (IQR)	Beta coefficient (CI)	Beta coefficient (CI)

Global Assessment of functioning — disability	65 (51–80)	48 (40–60)	81.5 (63.5–85.5)	12.78 (4.82 to 20.73)⁎⁎	− 10.16 (− 18.99 to − 1.33)⁎
	n (%)	n (%)	n (%)	OR (CI)	OR (CI)
Employment status:					
Employed 25–100%	14 (52.9)	36 (23.1)	23 (54.8)	–	–
Employed 0–25%	13 (48.2)	120 (76.9)	19 (45.2)	0.28 (0.12 to 0.65)⁎⁎	1.12 (0.43 to 2.96)
Main relationship status:					
Single/divorced/separated	14 (46.7)	130 (80.8)	22 (47.8)	–	–
In a relationship	16 (53.3)	31 (19.3)	24 (52.2)	4.79 (2.12 to 10.85)⁎⁎	1.05 (0.42 to 2.63)
Close confidant:					
No	2 (10.0)	43 (50.6)	9 (30.0)	–	–
Yes	18 (90.0)	42 (49.4)	21 (70.0)	9.21 (2.01 to 42.19)⁎⁎	3.86 (0.74 to 20.21)
Went to prison:					
No	31 (93.9)	132 (83.5)	46 (95.8)	–	–
Yes	2 (6.1)	26 (16.5)	2 (4.2)	0.33 (0.07 to 1.45)	1.48 (0.20 to 11.10)

Service use outcomes					

	Median (IQR)	Median (IQR)	Median (IQR)	Beta coefficient (CI)	Beta coefficient (CI)

Total number of days hospitalised	22 (0–99)	128 (48–371)	73 (28–165)	− 243.05 (− 337.01 to − 149.09)⁎⁎	− 54.72 (− 119.59 to 10.15)
	N (%)	N (%)	N (%)	OR (CI)	OR (CI)
Ever admitted compulsorily:					
No	16 (51.6)	37 (26.4)	12 (25.5)	–	–
Yes	15 (48.4)	103 (73.6)	35 (74.5)	0.34 (0.15 to 0.75)⁎	0.32 (0.12 to 0.84)⁎

OR = odds ratio; CI = 95% confidence interval; IQR = interquartile range; PMD = psychotic major depression; SZ = schizophrenia; BP = bipolar disorder.

⁎ p < 0.05.

⁎⁎ p < 0.01.

## References

[bb0005] Amin S., Singh S.P., Brewin J., Jones P.B., Medley I., Harrison G. (1999). Diagnostic stability of first-episode psychosis: comparison of ICD-10 and DSM-III-R systems. Br. J. Psychiatry.

[bb0010] Aronson T.A., Shukla S., Hoff A. (1987). Continuation therapy after ECT for delusional depression: a naturalistic study of prophylactic treatments and relapse. Convuls. Ther..

[bb0015] Aronson T.A., Shukla S., Hoff A., Cook B. (1988). Proposed delusional depression subtypes: preliminary evidence from a retrospective study of phenomenology and treatment course. J. Affect. Disord..

[bb0020] Baldwin P., Browne D., Scully P.J., Quinn J.F., Morgan M.G., Kinsella A., Owens J.M., Russell V., O'Callaghan E., Waddington J.L. (2005). Epidemiology of first-episode psychosis: illustrating the challenges across diagnostic boundaries through the Cavan-Monaghan study at 8 years. Schizophr. Bull..

[bb0025] Bromet E.J., Jandorf L., Fennig S., Lavelle J., Kovasznay B., Ram R., Tanenberg-Karant M., Craig T. (1996). The Suffolk County Mental Health Project: demographic, pre-morbid and clinical correlates of 6-month outcome. Psychol. Med..

[bb0030] Burns T., Creed F., Fahy T., Thompson S., Tyrer P., White I. (1999). Intensive versus standard case management for severe psychotic illness: a randomised trial. UK 700 Group. Lancet.

[bb0035] Ciccone J.R., Racy J. (1975). Psychotic depression and hallucinations. Compr. Psychiatry.

[bb0040] Ciompi L. (1980). Catamnestic long-term study on the course of life and aging of schizophrenics. Schizophr. Bull..

[bb0045] Cohen P., Cohen J. (1984). The clinician's illusion. Arch. Gen. Psychiatry.

[bb0050] Craig T.J. (1997). Diagnosis, treatment, and six-month outcome status in first-admission psychosis. Ann. Clin. Psychiatry.

[bb0055] Craig T.J. (2000). Is there an association between duration of untreated psychosis and 24-month clinical outcome in a first-admission series?. Am. J. Psychiatr..

[bb0060] Crebbin K., Mitford E., Paxton R., Turkington D. (2008). First-episode psychosis: an epidemiological survey comparing psychotic depression with schizophrenia. J. Affect. Disord..

[bb0065] DelBello M.P., Carlson G.A., Tohen M., Bromet E.J., Schwiers M., Strakowski S.M. (2003). Rates and predictors of developing a manic or hypomanic episode 1 to 2 years following a first hospitalization for major depression with psychotic features. J. Child Adolesc. Psychopharmacol..

[bb0070] Endicott J., Spitzer R.L., Fleiss J.L., Cohen J. (1976). The global assessment scale: a procedure for measuring overall severity of psychiatric disturbance. Arch. Gen. Psychiatry.

[bb0075] Harrison G., Hopper K., Craig T., Laska E., Siegel C., Wanderling J., Dube K.C., Ganev K., Giel R., an der Heiden W., Holmberg S.K., Janca A., Lee P.W., Leon C.A., Malhotra S., Marsella A.J., Nakane Y., Sartorius N., Shen Y., Skoda C., Thara R., Tsirkin S.J., Varma V.K., Walsh D., Wiersma D. (2001). Recovery from psychotic illness: a 15- and 25-year international follow-up study. Br. J. Psychiatry.

[bb0080] Heslin M., Lomas B., Lappin J., Donoghue K., Reininghaus U.A., Onyejiaka A., Croudace T., Jones P.B., Murray R.M., Fearon P., Dazzan P., Morgan C., Doody G.A. (2015). Diagnostic change ten years after a first episode of psychosis: findings from ÆSOP-10. Psychol. Med..

[bb0085] Hill M., Crumlish N., Clarke M., Whitty P., Owens E., Renwick L., Browne S., Macklin E.A., Kinsella A., Larkin C., Waddington J.L., O'Callaghan E. (2012). Prospective relationship of duration of untreated psychosis to psychopathology and functional outcome over 12 years. Schizophr. Res..

[bb0090] Holma K.M., Melartin T.K., Haukka J., Holma I.A.K., Sokero T.P., Isometsä E.T. (2010). Incidence and predictors of suicide attempts in DSM–IV major depressive disorder: a five-year prospective study. Am. J. Psychiatr..

[bb0095] Jager M., Bottlender R., Strauss A., Moller H.-J. (2005). Fifteen-year follow-up of Diagnostic and Statistical Manual of Mental Disorders, Fourth Edition depressive disorders: the prognostic significance of psychotic features. Compr. Psychiatry.

[bb0100] Kielhorn J.M., Graf vonSchulenberg A. (2000). The Health Economics Handbook.

[bb0105] Kirkbride J.B., Fearon P., Morgan C., Dazzan P., Morgan K., Tarrant J., Lloyd T., Holloway J., Hutchinson G., Leff J.P., Mallett R.M., Harrison G.L., Murray R.M., Jones P.B. (2006). Heterogeneity in incidence rates of schizophrenia and other psychotic syndromes: findings from the 3-center ÆSOP study. Arch. Gen. Psychiatry.

[bb0110] Kirkbride J.B., Errazuriz A., Croudace T.J., Morgan C., Jackson D., Boydell J., Murray R.M., Jones P.B. (2012). Incidence of schizophrenia and other psychoses in England, 1950–2009: a systematic review and meta-analyses. PLoS One.

[bb0115] Mallett R. (1997). Sociodemographic Schedule.

[bb0120] Morgan C., Lappin J., Heslin M., Donoghue K., Lomas B., Reininghaus U.A., Onyejiaka A., Croudace T., Jones P.B., Murray R.M., Fearon P., Doody G.A., Dazzan P. (2014). Reappraising the long-term course and outcome of psychotic disorders: the ÆSOP-10 study. Psychol. Med..

[bb0125] Opjordsmoen S. (1989). Long-term course and outcome in unipolar affective and schizoaffective psychoses. Acta Psychiatr. Scand..

[bb0130] Radomsky E., Haas G., Mann J., Sweeney J. (1999). Suicidal behavior in patients with schizophrenia and other psychotic disorders. Am. J. Psychiatr..

[bb0135] Sands J.R., Harrow M. (1999). Depression during the longitudinal course of schizophrenia. Schizophr. Bull..

[bb0140] Sartorius N., Gulbinat W., Harrison G., Laska E., Siegel C. (1996). Long-term follow-up of schizophrenia in 16 countries. A description of the international study of schizophrenia conducted by the World Health Organization. Soc. Psychiatry Psychiatr. Epidemiol..

[bb0145] Schimmelmann B.G., Conus P., Edwards J., McGorry P.D., Lambert M. (2005). Diagnostic stability 18 months after treatment initiation for first-episode psychosis. J. Clin. Psychiatry.

[bb0150] StataCorp LP (2009). STATA 10.1 for Windows.

[bb0155] Stephens J.H. (1982). A comparison of nine systems to diagnose schizophrenia. Psychiatry Res..

[bb0160] Takei N., Persaud R., Woodruff P., Brockington I., Murray R.M. (1998). First episodes of psychosis in Afro-Caribbean and White people. An 18-year follow-up population-based study. Br. J. Psychiatry.

[bb0165] Tsuang D., Coryell W. (1993). An 8-year follow-up of patients with DSM-III-R psychotic depression, schizoaffective disorder, and schizophrenia. Am. J. Psychiatr..

[bb0170] Warner R., Morgan C., McKenzie K., Fearon P. (2008). Social Factors as a Basis for Treatment in Society and Psychosis.

[bb0175] Welner A., Croughan J., Fishman R., Robins E. (1977). The group of schizoaffective and related psychoses: a follow-up study. Compr. Psychiatry.

[bb0180] Whitty P., Clarke M., McTigue O., Browne S., Kamali M., Larkin C., O'Callaghan E. (2005). Diagnostic stability four years after a first episode of psychosis. Psychiatr. Serv..

[bb0185] Winokur G., Black D.W., Nasrallah A. (1992). The Schizoaffective Continuum.

[bb0190] World Health Organisation (1993). The ICD-10 Classification of Mental and Behavioural Disorders: Diagnostic Criteria for Research Geneva.

[bb0195] World Health Organisation (1994). SCAN V2 (Schedules for Clinical Assessment in Neuropsychiatry: Version 2).

